# Small Molecule Radiopharmaceuticals – A Review of Current Approaches

**DOI:** 10.3389/fmed.2016.00005

**Published:** 2016-02-23

**Authors:** Shubhra Chaturvedi, Anil K. Mishra

**Affiliations:** ^1^Division of Cyclotron and Radiopharmaceutical Sciences, Institute of Nuclear Medicine and Allied Sciences, Defence Research and Development Organisation, Delhi, India

**Keywords:** radiopharmaceuticals, multivalent ligands, bioorthogonal approaches, surface modification, cross-coupling reaction

## Abstract

Radiopharmaceuticals are an integral component of nuclear medicine and are widely applied in diagnostics and therapy. Though widely applied, the development of an “ideal” radiopharmaceutical can be challenging. Issues such as specificity, selectivity, sensitivity, and feasible chemistry challenge the design and synthesis of radiopharmaceuticals. Over time, strategies to address the issues have evolved by making use of new technological advances in the fields of biology and chemistry. This review presents the application of few advances in design and synthesis of radiopharmaceuticals. The topics covered are *bivalent ligand approach and lipidization* as part of design modifications for enhanced selectivity and sensitivity and *novel synthetic strategies* for optimized chemistry and radiolabeling of radiopharmaceuticals.

## Introduction

Radiopharmaceuticals are being used in diagnostics and therapeutics for more than half a century. They are widely used in the delineation of neurodegenerative diseases, myocardial imaging and diagnosis, and treatment of cancer. Due to their wide application, the development of an “ideal” radiopharmaceutical continues to be the foremost challenge of the research frontier in nuclear medicine. The key issues confronting the research community in radiopharmaceutical chemistry is to develop highly specific and selective ligands with high specific activity capable of targeting and overcoming biological barriers.

The challenges emanate at the different stages of developing radiopharmaceutical, *viz*., design, modification, and radiolabeling. Selection of the type of molecule (antibody and their fragments, peptides, nucleosides, aptamers, small molecules), surface modifications, multivalency, and labeling reactions optimization are few variations that have been used to address the challenges. Based on these variations, the review presents three emerging approaches that address the challenges: high selectivity and sensitivity through design optimization using bivalent ligands (BLs), targeting against natural barriers through modification using lipidization, and high specific activity while radiolabeling using sophisticated chemistries, *viz*., bioorthogonal and cross-coupling reactions. These approaches have the potential to be integrated into radiopharmaceutical development. We describe each of these approaches *seriatim* in along with avenues for future research in Sections 1–3.

## 1 High Selectivity through Bivalent Ligand Approach

### Bivalent Ligand Approach

**Figure 1:**
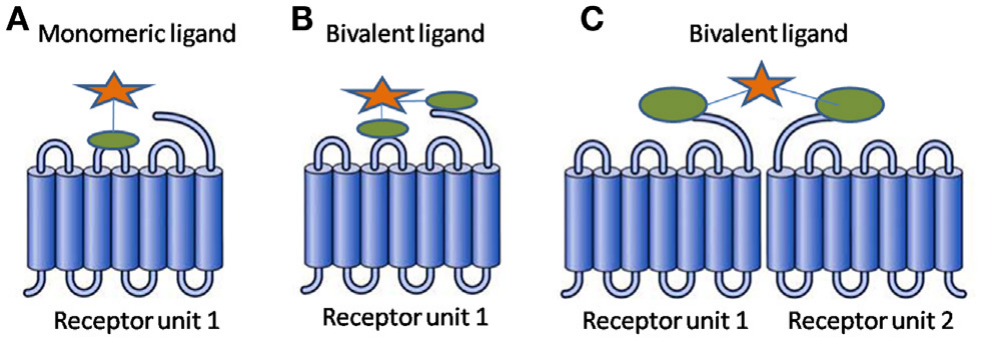
**Binding modes for a ligand (A) monovalent ligand with one receptor unit (B) bivalent ligand with one receptor unit (C) bivalent ligand with receptor dimer**.

In simplest terms, a BL consists of two pharmacophores linked through a spacer. The two pharmacophores can be identical resulting in a homobivalent ligand or different resulting in a heterobivalent ligand. The BL benefits from the collaborative binding of the two pharmacophores, resulting in favorable thermodynamics as compared to that of a monovalent ligand (1). Figure 1 presents binding modes a BL can exhibit.

### Selectivity through BLA

Bivalent ligands are examples of multimeric interactions. Multimeric interactions are known to enhance the binding affinity of the ligands through multiple mechanisms, e.g., receptor clustering, chelating effect on receptors, ligand–receptor steric stabilization, and ligand accumulation near the receptor (2). Overall, the effect is enhanced selectivity and enhanced binding affinity (1). The multivalent concept has been extensively validated for peptides. Successful reports for multimeric peptides as diagnostics agent are included in Table 1.

**Table 1:** **Few reports on successful targeting using multimeric peptides**.

Multimeric RGD as integrin αvβ3 targeting unit conjugated to acyclic and cyclic chelators with dimeric, tetrameric, and octameric units	(3–5)
Heterobivalent peptides SPECT imaging agent for neutrophilic inflammation	(6)
Melanocortin receptors peptide-based ligands	(7)
Bombesin peptide	(8)

Reviews regarding the development of homo-multimeric and hetero-multimeric peptidic ligands are many, and hence, for peptidic multimeric ligands readers may refer reviews (4, 9). The multimeric concept is now being extended to small molecules as well. Small molecule-based BLs are capable of multimeric interactions, thereby having higher sensitivity and selectivity.

### Applications of BLA

A BL functions best when multiple binding pockets are present in the target. Depending on the pharmacophores, a BL can target one or multiple biomarkers. Tumor targeting can benefit from the high binding avidity and selectivity of BL. Furthermore, hetero-BL can result in more specificity as it targets different receptors simultaneously.

Receptor-based imaging, especially for neuroreceptors, can also benefit from the bivalent approach. Many receptors/neuroreceptors belong to G-protein coupled receptor (GPCR) family (10). After the reports about the existence of GPCRs as oligomers and higher-orders started pouring (11), BLs were successfully developed and validated against them. The approach has been of high relevance in the design and development of second generation antipsychotics (12, 13). A BL can target both homo- and hetero-dimeric receptor systems depending on the pharmacophores.

Another target for BLs is β-amyloid plaques because of the presence of multiple binding sites (14).

### Development Considerations for BLA

The key factors for BL design are (a) selection of pharmacophores, (b) optimization of linker length and its biocompatibility, and (c) spatial parameters of the final compound (2). As a radiopharmaceutical, a BL has to be evaluated for its *in vitro* and *in vivo* properties.

**Figure 2:**
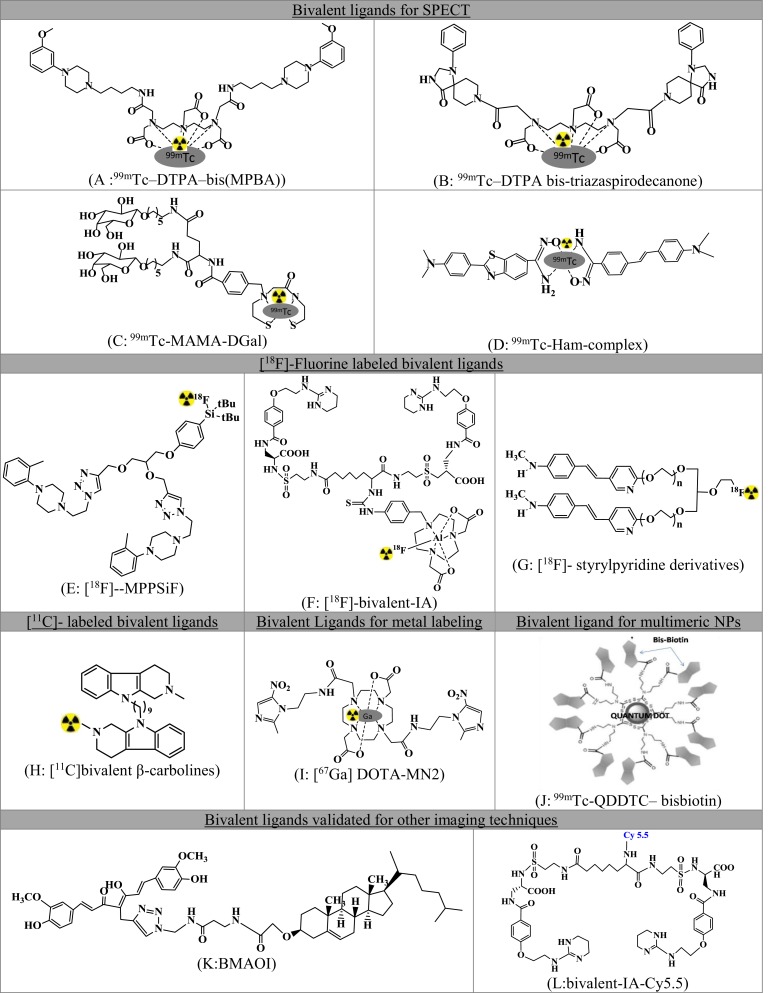
**Comprehensive list of small molecule-based bivalent ligands for diagnostics**. **(A)** ^99m^Tc-DTPA-bis(MPBA), **(B)** ^99m^Tc-DTPA bis-triazaspirodecanone, **(C)** ^99m^Tc-MAMA-DGal, **(D)** ^99m^Tc-Ham, **(E)** [^18^F]-MPPSiF, **(F)** [^18^F]-bivalent-IA, **(G)** [^18^F]-styrylpyridine derivatives, **(H)** [^11^C]bivalent β-carbolines, **(I)** [^67^Ga]DOTA-MN2, **(J)** ^99m^Tc-QDDTC-bisbiotin, **(K)** BMAOI, and **(L)** bivalent-IA-Cy5.5.

A series of small molecule-based dimeric and multimeric ligands have been developed and reported in recent past for targeted imaging of tumors, receptors, and β-amyloid plaques. Figure 2 summarizes radiolabeled small molecule-based BLs.

### Bivalent Ligands Demonstrated for SPECT

#### Receptor Imaging

Singh et al. (15) demonstrated the proof-of-concept for 5HT_1A_ receptors using homodimeric ligand and validated the ligand as a SPECT imaging agent. Two identical pharmacophores based on 1-(2-methoxyphenyl)piperazine (MPP) were linked using an aliphatic linker of four carbon atoms to the acyclic chelating agent DTPA and validated as SPECT agent after technetium labeling [^99m^Tc-DTPA-bis(MPBA) Figure 2A]. The authors were able to demonstrate (a) 1000 times high selectivity toward 5HT_1A_ receptors than 5HT_2A_ receptors, (b) involvement of both the pharmacophores for bivalent binding using hill slope analysis, and (c) high labeling efficiency.

On similar lines, using DTPA as an acyclic chelator for technetium (16), reported the synthesis of bis-triazaspirodecanone (Figure 2B). The ligand showed enhanced binding affinity theoretically using docking and MM-GBSA calculations. Furthermore, the compound showed selective striatum uptake in the brain and selective dopamine D2 targeting.

Similarly, the divalent ligand with two units of galactose derivatives (^99m^Tc-MAMA-DGal, Figure 2C) showed higher specific binding to asialoglycoprotein receptors (ASGPR) in dynamic microSPECT imaging and biodistribution studies of liver fibrosis (17). The monovalent ligand ^99m^Tc-MAMA-MGal was also validated for comparison. The divalent ligand showed better binding affinity *in vitro* and fast pharmacokinetics.

#### β-Amyloid Imaging

To assess the amyloid aggregation (18), synthesized bivalent amyloid ligand and labeled with ^99m^Tc leading to the formation of ^99m^Tc-Ham (Figure 2D). Stilbene (SB) and benzothiazole (BT) derivatives were selected as amyloid binding units. These were conjugated to a hydroxamamide (Ham) and labeled for SPECT imaging using ^99m^Tc. Five analogs were synthesized and evaluated for binding affinity and brain uptake.

### [^18^F]-Fluorine-Labeled Bivalent Ligands

#### Receptor Imaging

In another study of Hazari et al. (19), bis-MPP (Figure 2E) derivative has been synthesized to image serotonin receptors. The duplication of the pharmacophores leads to a supra-additive increase in binding and potency as compared to monovalent analog. Thus, the bis-compound had sub-nanomolar affinity for the receptor, 1000 times more selectivity for 5HT_1A_ as compared to D_2_, 5-HTT, or 5HT_2A_. The compound was validated as PET imaging agent.

For the imaging of αvβ3, a non-peptidic BL was reported by Wang et al. (20) (Figure 2F). This molecule consisted of two units of antagonist 4-[2-(3,4,5,6-tetrahydropyrimidine-2-lamino)-ethyloxy]benzoyl-2-(S)-aminoethylsulfonyl-amino-h-alanine (IA) and radiolabeled using ^18^F-AlF/NODA chelation reaction.

#### β-Amyloid Imaging

A series of bivalent (Figure 2G) and trivalent ^18^F-styrylpyridine derivatives were developed for imaging β-amyloid plaques in the brain. The BL displayed high binding affinity. The study demonstrated the effect of linkers and the geometry of the molecule on the binding affinity. An ether linkage was found to have higher binding affinity *vis*-*à*-*vis* an amide linkage. The trivalent molecule had a reduced binding affinity as compared to the BL (14).

### [^11^C]-Labeled Bivalent Ligands

#### Enzyme Imaging

β-Carboline bivalent derivatives that are known inhibitors for acetylcholinesterase (AChE) and butyrylcholinesterase (BChE) were developed for imaging of cholinesterase in Alzheimer’s disease. The derivatives were radiolabeled at the nitrogen position of the amine precursor through *N*-[^11^C]methylation using [^11^C]CH_3_I (Figure 2H). Radiolabeling parameters of three derivatives of variable linker length were reported (21).

### Bivalent Ligands for Metal Labeling

#### Tumor Imaging

Bivalent ligand concept has also been validated for metal-based radiopharmaceuticals. Metronidazole was conjugated to DOTA (DOTA-MN2, Figure 2I) and developed as radiogallium–DOTA complex without reducing the radiogallium complex stability for the imaging of hypoxic lesions using PET/SPECT (22). The complex showed significant tumor uptake and low non-target accumulation.

### Bivalent Ligand for Multimeric Nanoparticles

#### Tumor Imaging

The concept of enhanced binding *via* multivalency using small molecules and nanoparticles (NPs) has also been reported. Nanoparticles (Quantum dots), as reported in the work of Bag et al. (23), were conjugated with multiple biotin units (bisbiotin) to have enhanced selectivity.^99m^Tc-QDDTC-bisbiotin showed significantly higher tumor uptake, better tumor retention, and enhanced pharmacokinetics as compared to DTC–bisbiotin ligand. The work illustrates the bivalent effect of bisbiotin ligand for high tumor uptake. Other effects, *viz*., better tumor retention and enhanced pharmacokinetics were the results of the enhanced permeable and retention (EPR) effect due to the QD (Figure 2J).

### Potential Bivalent Ligands Validated for Other Imaging Techniques

#### β-Amyloid Imaging

Though not as a radiopharmaceutical, amyloid-β plaque imaging was accomplished using curcumin and cholesterol BL (BMAOI, Figure 2K), which could bind to various Aβ42 species with micromolar binding affinity and has appropriate fluorescence properties for labeling and imaging Aβ plaques *in situ* (24).

#### Receptor Imaging

NIR imaging probe for αvβ3 (25): Figure 2L was reported for cancer imaging. The non-peptidic small molecule bivalent antagonist demonstrated improved binding avidity relative to the monovalent ligand.

### Bivalent Ligands for Radiotherapy

As above-mentioned BLs alone are being used for the development of atypical antipsychotics. Bivalent peptide-based ligands are reported for radiotherapy applications (9). However, to the best of our knowledge, examples of small molecule-based BLs for radiotherapy have not been reported.

#### Future Directions

The advantages of high sensitivity, selectivity, and favorable pharmacodynamics make radiolabeled BLs promising candidates for diagnostics and possibly therapy. However, knowledge gaps in receptor expression patterns, receptor’s higher order structures, and binding pattern on receptors need to be filled for full utilization of the approach. In terms of ligands itself, an exact mechanistic aspect of the binding of ligand need to be understood. The structural features, pharmacophore, the cooperative effect on the binding of pharmacophore, linker length, and geometry effect all have to be considered in the design of the ligand. Such studies can take lead from theoretical screening-like docking and high-throughput screening or through control experiments, which include comparative studies with a monovalent ligand. The approach still needs to be extended to radiotherapy.

The radiolabeled BLs are promising candidates in diagnostics and can enhance the binding affinity and enable multi-targeting. However, the penetration ability across the cellular membrane and the circulation time that determines the serum availability of the radiopharmaceutical are also important for the efficacy. The following section discusses the efforts in delivering the radiopharmaceutical to the target site through lipidization and surface modification.

## 2 Enhanced Targeting through Lipidization and Surface Modification

### Lipidization

Lipidization is a chemical approach to alter the solubility and pharmacokinetic behavior of a molecule. It involves attachment of lipid at the polar end of a molecule, thereby conferring lipophilic nature to the molecule.

### Enhanced Targeting through Lipidization

Lipidization of drugs in the form of (a) Prodrug Strategy and (b) lipid-based carriers’ *viz*. Liposomes and lipidized NPs can enhance the drug targeting. This is because of (a) enhanced permeability across biological barriers, namely, the membranes, (b) improved pharmacokinetics that includes enhanced circulation time, (c) slow release, thereby prolonging drug action, and (d) enhanced bioactivity through passive targeting. This approach has been used for developing anticancer drugs, drugs for liver diseases, and the lymphatic system. The strategy can also provide a solution for CNS targeting due to BBB penetration (26).

### Lipidic Prodrugs for Imaging/Radiopharmaceuticals

**Figure 3:**
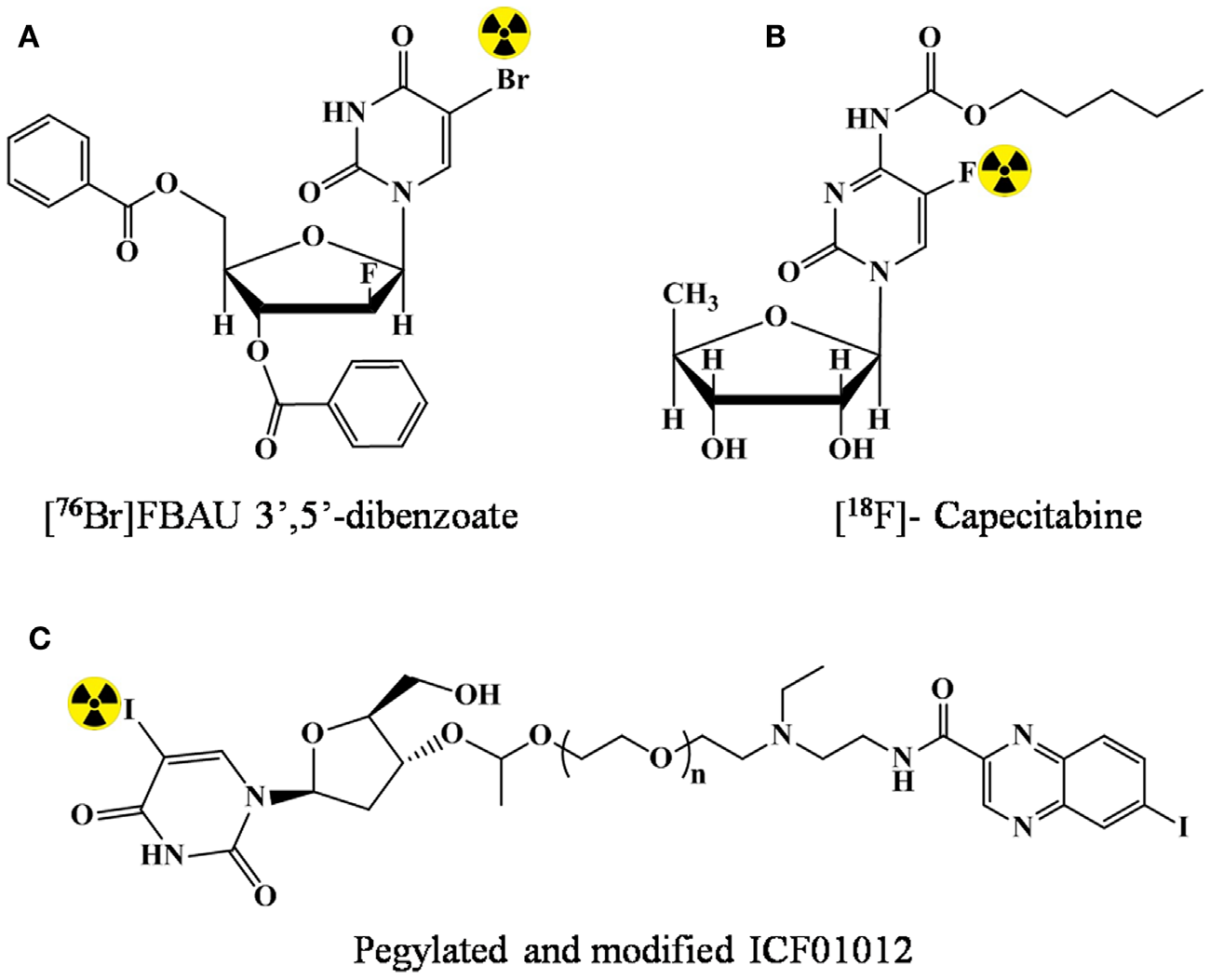
**Lipidic modification of the nucleosides for prodrug strategy**. **(A)** [^76^Br]FBAU 3′,5′-dibenzoate, **(B)** [^18^F]-Capecitabine, and **(C)** pegylated and modified ICF01012.

The lipidic modification can lead to enhanced permeability in the brain, and hence, has potential for brain imaging. However, in literature, examples highlighting the utility of lipidic prodrug for imaging are rare. In 2002, Kao et al. (27) demonstrated that an additional lipophilic character by benzoylation at 3′ and 5′ of FBAU enhanced the uptake in brain having normal blood–brain barrier. The prodrug FBAU 3′,5′-dibenzoate was radiolabeled with ^76^Br. Biodistribution studies indicated a higher brain accumulation of radioactivity (up to two times) at all time points in rats injected with [^76^Br]FBAU 3′,5′-dibenzoate (Figure 3A) than with [^76^Br]FBAU.

In 2005 (28), in order to reduce the toxicity and enhance the tumor penetration capability of 5-FU, prodrug strategy was validated. Capecitabine (*N*^4^-*n*-pentyloxycarbonyl-5′-deoxy-5-fluorocytidine), which happened to be the first and the only orally administered fluoropyrimidine approved for the use as a second-line cancer therapy was labeled with ^18^F (Figure 3B). However, the study only included radiolabeling optimization, and no data for the capability of enhanced penetration/reduced was presented. In a present study of André et al. (29), *N*,*N*-diethyl *N*,*N*-diethylaminoethyleneheteroarylamide derivatives (e.g., ICF01012) was pegylated and conjugated with anti-metabolite 5-iodo-2′-deoxyuridine (IUdR). Enhanced and prolonged tumor uptake (melanoma) was observed after radiolabeling with ^125^I (Figure 3C).

### Lipidic Nanoparticles for Imaging/Radiopharmaceuticals with Surface Modification

Lipidization can lead to enhanced efficiency of drug delivery systems. Lipid-based NPs consist of two types (a) liposomes and (b) solid lipid NPs (30). Encapsulation of drugs in these NPs protect from hydrolysis and aid in sustained controlled release at the site of interest. Reviews regarding lipid-based NPs are listed in Table 2. When radiolabeled the liposomes and NPs can prove to be effective theranostic agents.

**Table 2:** **Reviews regarding lipid-based nanoparticles**.

Nanoparticles for brain drug delivery	(31)
• Liposomes (lipid-based nanoparticles)	
• Polymer-based nanoparticles (polymeric nanoparticles, polymeric micelles, dendrimers)	
New developments in liposomal drug delivery	(32)
• Synthesis	
• Targeting strategies	
• Variations of nanoparticles	
• Applications	
Emerging role of radiolabeled nanoparticles as an effective diagnostic technique	(30)
• All major classes of nanoparticles and their utilization in imaging	
Lipid- and polymer-based nanostructures for cancer theranostics	(33)
• Application of nanoparticles for cancer theranostics and imaging	
Nanoparticle PEGylation for imaging and therapy	(34)

Liposomes are further surface modified for both enhanced pharmacokinetics and enhanced penetration. The modifications can include pegylation, squalenolation, and peptidization.

#### Pegylation

For surface modification of NPs (liposomes), pegylation is one of the most successful strategies. Pegylation is known to enhance the circulation time for NPs. Few examples of pegylation, especially in context with radio imaging are being discussed covering the following aspects:

(a) Pegylated liposomes with enhanced pharmacokinetics for imaging
(b) Pegylated liposomes with enhanced BBB permeation and with enhanced pharmacokinetics for imaging.

##### Pegylated Liposomes with Enhanced Pharmacokinetics for Imaging

###### Pegylated Nucleolipids for Imaging with Improved Pharmacokinetics

**Figure 4:**
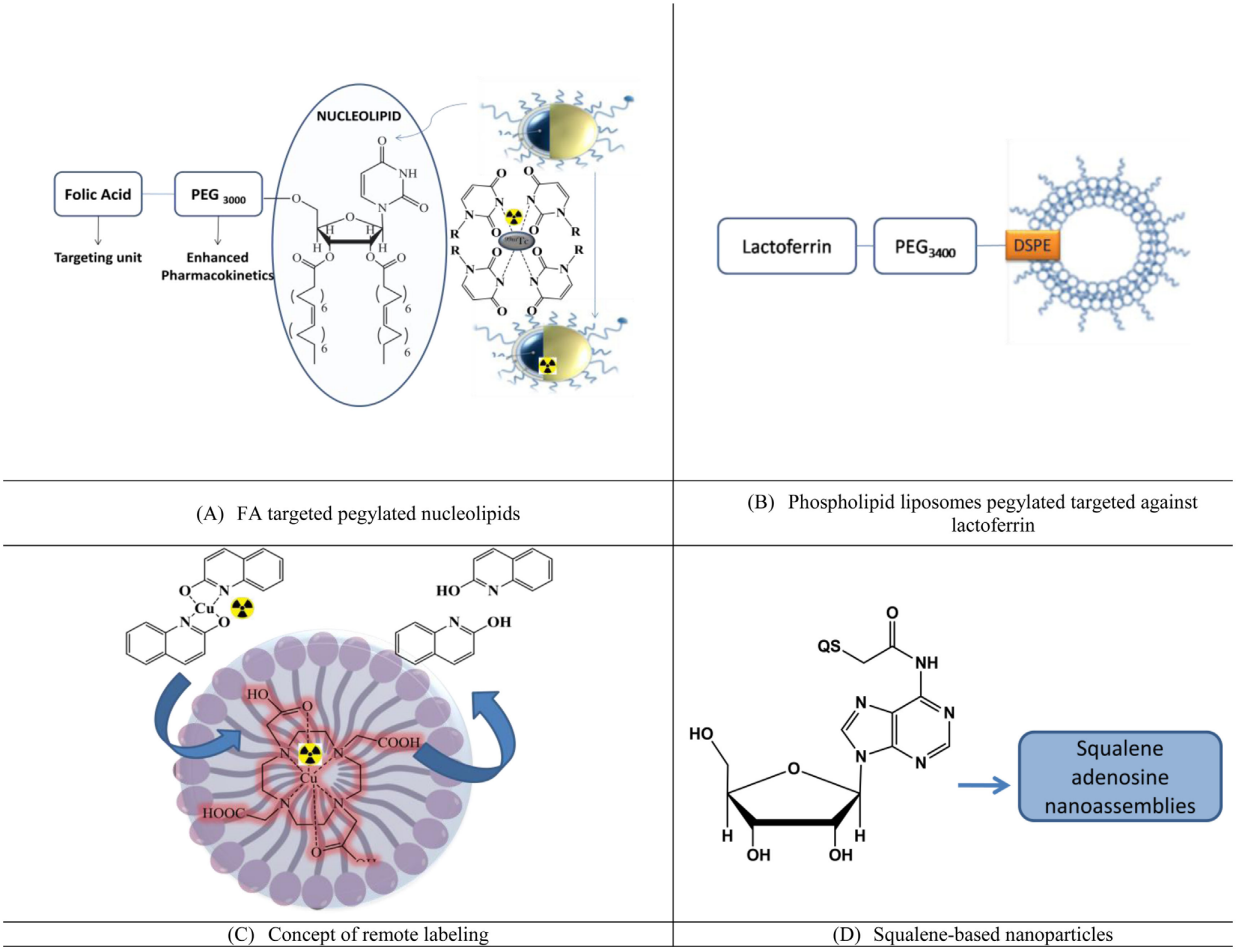
**Lipidic nanoparticles for imaging and enhanced pharmacokinetics**. **(A)** FA targeted pegylated nucleolipids, **(B)** phospholipid liposomes pegylated targeted against lactoferrin, **(C)** concept of remote labeling, and **(D)** squalene-based nanoparticles.

Nucleolipids are an emerging class of drug delivery systems. Recently, liposomes using the hybrid nucleoside lipids (NLs) were developed in which nucleosides were pegylated and targeted against folic acid. These liposomes (Figure 4A) were developed as the theranostic agent by encapsulating cisplatin as the therapeutic agent and ^99m^Tc radiolabeled using the uridine rings at the outer surface of the liposomes. Enhanced uptake at the tumor site was observed along with the favorable pharmacokinetics, which included enhanced circulation time (35).

##### Pegylated Liposomes with Enhanced BBB Permeation with Enhanced Pharmacokinetics

###### Pegylated Phospholipidic

Lactoferrin targeted pegylated phospholipdic liposomes (Lf-PL-^99m^Tc) based on distearoylphosphatidylcholine (DSPC), cholesterol, and distearoylphosphatidylethanolamine were radiolabeled and evaluated for BBB penetration and effect on pharmacokinetics [(36), Figure 4B].

1. BBB penetration: bEnd.3 cells, which is an immortalized mouse brain endothelial cell line was used as a mimic for BBB. The cellular uptake was significantly higher for the targeted liposome. Biodistribution studies indicated an enhanced uptake of the lipidic liposomes, which were targeted with lactoferrin, and approximately 1.47 times more uptake was reported than the non-targeted pegylated liposomes. However, the study did not comment on the penetration ability due to pegylation.
2. Pharmacokinetics: the area under the curve (AUC_0 → 24 h_) and the clearance rate (Cl) from Lf-PL-^99m^Tc was found to be similar to PL-^99m^Tc with *p*-values of 0.89 and 0.31, respectively. Thus, the Lf-conjugated liposomes could provide the similar long-circulation property *in vivo*. For designing a better Lf-PL-^99m^Tc, the number of Lf ligand on the liposomes should have a suitable level.

Several other targeted pegylated liposomal preparations have also been reported (34).

Pegylated liposomes consisting of 1,2-distearoyl-sn-glycero-3-phosphocholine (DSPC), cholesterol (Chol), and 1,2-distearoyl-sn-glycero-3-phosphoethanolamine-*N*-[methoxy (polyethylene glycol)-2000] (DSPE-PEG2000) were used for remote loading of radionuclide. In the work of Petersen et al. (37) (Figure 4C), [64]Cu was crossed across the membrane of preformed liposomes into the aqueous cavity using a new ionophore, 2-hydroxyquinoline, in order to achieve high and stable loading of radionuclides.

#### Squalenolation

Though not with liposomes, squalene adenosine nano-assemblies (SqAdNA) were studied for their interaction with endothelial cells of the human brain to assess the mechanism of penetration (38). The internalization was mainly mediated by the LDL receptors-mediated endocytosis, after which the NA disassembled inside the cells and exocytosed as single molecules. Such assemblies were also prepared with an array of nucleosides (deoxycytidine-Sq, thymidine-Sq, gemcitabine MP-Sq, ddI-Sq, and deoxycytidine-5′-Sq) and studied to assess the influence of the nucleoside nature and position with respect to squalene on the structure of the NAs (39), Figure 4D. However, the utilization of the assemblies for imaging and brain penetration *in vivo* is yet to be validated.

#### Peptidization

Liposomal vector was modified as a novel bi-ligand having transferrin for targeting and poly-l-arginine for enhanced uptake in the brain (40). The bi-ligand liposomes accumulated in the rat brain at significantly (*p* < 0.05) higher concentrations as compared to the single-ligand (transferrin) or plain liposomes.

##### Future Directions

The prodrug approach needs to be exploited for design of lipidic-based radiopharmaceutical. From design perspective choice of lipids can be important for effectiveness. Literature available till date does highlight features of fatty acids (FA) required for effectiveness. For example, the importance of carboxylate in cellular internalization, an effect of chain length (longer chain fatty acid are more stable than shorter chain FA and better suited for lymphatic targeting) and pros and cons when exploiting carboxylate or ω-position for drug conjugation. Further studies on the effect of chain length of FA on targeting the type of membrane will be helpful. Prodrugs whether radiolabeled or not, also suffer from one challenge, guarantee of conversion from inactive to active form in the living system.

The drug delivery systems, liposomes and solid lipid NPs are expensive options with limited shelf life. Second, their toxicity, especially for cationic liposomes, and cellular interaction need to be addressed. Future work needs to address the issues for successful utilization of liposomes and solid lipid NPs as drug delivery systems and radiopharmaceuticals.

The concluding step in the synthesis of any radiopharmaceutical is the radiolabeling. A molecule with good selectivity and sensitivity and also with good penetration ability may not prove to be an ideal radiopharmaceutical because of the poor specific activity after radiolabeling. Hence, novel and optimized radiolabeling conditions play an important role in the development of a radiopharmaceutical. A lot of work has been done in this regard. The following section gives an overview of the development in radiolabeling chemistry.

## 3 Synthesis and Radiolabeling Optimization

### Radiolabeling

Radiolabeling is the incorporation of the radioactive moiety in a compound in order to track the compound. With the growing utilization of diverse molecules as radiotracers, there is a growing need for new or modified radiolabeling methods that require low quantities of bioactive compounds, employ mild conditions to avoid loss of bioactivity, have short reaction times for short-lived radionuclides, and result in high specific activity. At the same time, for human application, the new or modified radiolabeling methods need to focus on toxic free reagents or supplemented with better purification procedures. Bioorthogonal and cross-coupling are upcoming approaches in order to meet the above requirements.

Radiolabeling can proceed in two ways: (a) using radiolabeled prosthetic groups that are coupled to bioactive molecules using bioorthogonal reactions and (b) direct labeling of bioactive molecules using cross-coupling reactions.

### Bioorthogonal Approaches

Bioorthogonal reactions can proceed in the living systems without influencing or getting influenced by the biological processes, the efficacy of the ligands is retained and can demonstrate fast kinetics especially when used for monitoring.

It may be noted that a large number of reviews have already been published, which cover the detailed aspects (41, 42). Hence, here a summarization along with few additions is being given for different types of bioorthogonal approaches.

#### Copper-Based Click Ligation

*Click chemistry* as described by K. Barry Sharpless is “a set of powerful, virtually 100% reliable, selective reactions for the rapid synthesis of new compounds” (29, 43). Click chemistry reports in radiopharmaceutical sciences were first published in 2006 (44). It has been extensively studied and published. Many comprehensive reviews are available. Some examples are (a) click chemistry mechanism (45), (b) application in radiopharmaceuticals (43, 44), (c) application with specific precursors-glycobiology (46), (d) click chemistry in chelate development (44), and (e) patent analysis (47).

An overview of click chemistry for radiopharmaceuticals is as follows:

(a) Due to its bioorthogonal nature, click chemistry has been widely applied with different types of precursors.
(b) Its application extends from
  - Linking two biomolecules without compromising the bio-efficacy
  - Developing prosthetic groups that serve as radiolabeling precursors for fluorine-18 and carbon-11. Choice of a prosthetic group can influence (a) metabolic profile (b) *in vivo* behavior (41).
  - Novel chelate development wherein the triazole moiety acts as an electron donor to the metal.

**Figure 5:**
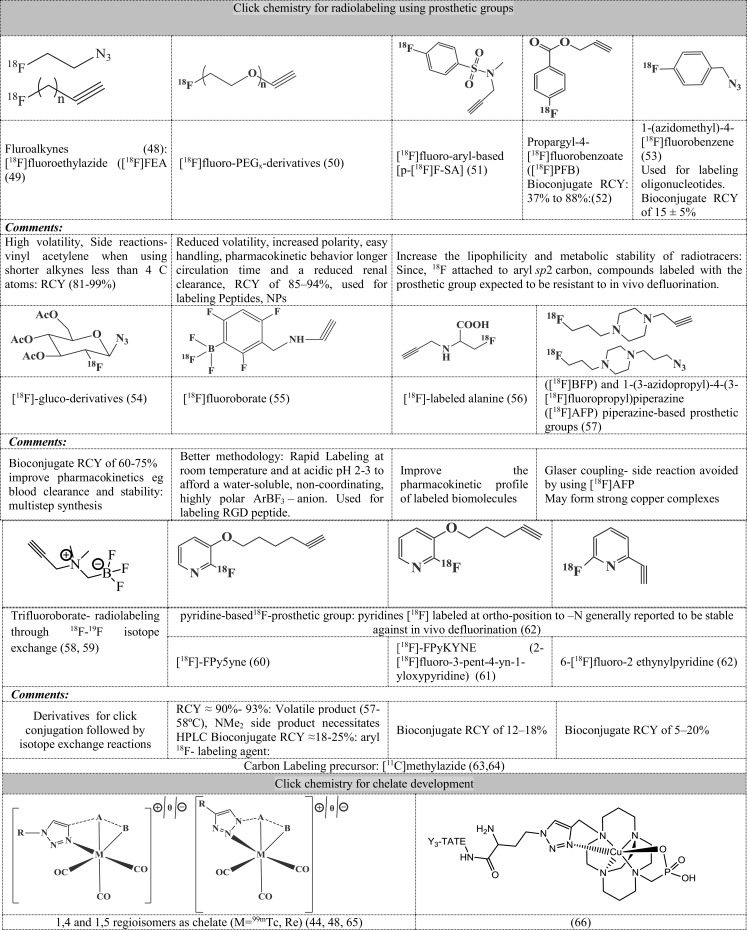
**Applications of click chemistry**. Structures referenced in (41, 42, 48–66).

Few representative structures developed using click chemistry are shown in Figure 5 [structures referenced in Kettenbach et al. (41) and Pretze et al. (42)] covering the aspects b (ii) and b (iii). Though most popular as copper (I) catalyzed click chemistry leading to selective formation of 1,4 regioisomer, another variation using ruthenium complexes which leads to selective 1,5 regioisomer has also been explored.

#### Strain-Promoted Click Chemistry/Strain-Promoted Azide Alkyne Cycloaddition

**Figure 6:**
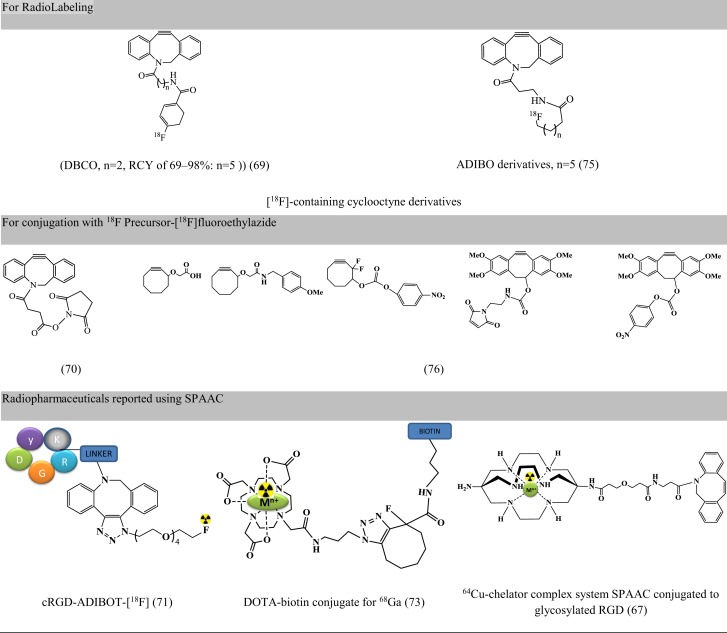
**Representative examples for radiopharmaceuticals using SPAAC**. Structures referenced in (42, 67, 69–71, 73, 75, 76).

Largely driven by the requirement of copper-free click chemistry due to copper linked toxicity (cytotoxicity, non-compatibility with oligonucleotides, hepatitis, and implications in Alzheimer’s disease and neurological diseases), strain-promoted, and copper-free variants of click chemistry are being validated in radiopharmacy (42). Apart from being copper-free, the reaction proceeds at a faster rate and can be used for short-lived radioisotopes like ^64^Cu (67); it is efficient, has high specificity, and requires mild reaction conditions (68). These were first reported in 2011 (69). Since then, the reaction has been used for radiolabeling of peptides [BBN (70), RGD (67, 71), c-Met-binding peptide (71), apoptosis-targeting peptide (ApoPep) (72), somatostatin analogs (72), DOTA-biotin conjugate (73), and NPs (68, 74)]. However, the concern for strain-promoted azide alkyne cycloaddition (SPAAC) include (a) effect of bulky moieties such as DBCO and ADIBO on lipophilicity, binding affinity with the target and the variation on pharmacokinetic behavior, and (b) non-regioselective product formation consisting both 1,4 and 1,5 regioisomers (72). Figure 6 presents precursors for fluorine labeling and radiopharmaceuticals developed using SPAAC.

#### Other Ligations: Staudinger Ligation, Tetrazines (Tetrazine-Trans-Cyclooctene Ligation), and Radio-Kinugasa Reaction

##### Staudinger Ligation

Staudinger ligation is another example of the metal-free conjugation reaction (42). Two variants exist: the non-traceless with the inclusion of phosphine oxide and the traceless version without the inclusion of the phosphine oxide in the final product. Both lead to the formation of the amide bond. The non-traceless version has not been as widely applied as the traceless version. Furthermore, the reaction can be accomplished either through direct approach (azide of biomolecule reacted with ^18^F-phosphanes) or indirect approach (phosphane derivatized biomolecule reacted with ^18^F-azide). The range of radiopharmaceuticals developed using the Staudinger Ligation is covered in the review (42).

##### Tetrazines (Tetrazine-Trans-Cyclooctene Ligation) (41, 42)

Tetrazine-trans-cyclooctene ligation (TTCO ligation), introduced in 2010, is the inverse electron demand of the Diels–Alder (IEDDA) cycloaddition between a cyclooctene and a 1,2,4,5-tetrazine under the release of nitrogen (77). Here, the tetrazine functionalized biomolecule is reacted with an ^18^F-labeled cyclooctene (more preferred for radiolabeling). The approach has the advantages of fast reaction rates even without catalyst making it suitable for ^11^C-labeling reaction (78), non-reversibility because of nitrogen release, broad tolerance range, both aqueous and organic based high yields. Its mechanism and the application are covered in the review (42). In short, the reaction has been applied for labeling peptides (RGD, GLP-1, exendin), small molecules PARP1-targeting small molecule and DOTA derivatives [refer review (79)].

##### Radio-Kinugasa Reaction

A recent addition to the radio fluorination is the Kinugasa reaction validated in 2014 (80). Advantage includes fast kinetics and a broad spectrum of biological activities and low toxicity of β-lactams. Radiochemical yields of the Kinugasa reaction products could be significantly increased by the use of different Cu(I) ligands (81).

### Novel Cross-Coupling Approaches

The transition metal-mediated cross-coupling reactions have been used as part of organic synthesis for the precursors for radiolabeling. The cross-coupling reactions came into the picture in 1995 with the work of Langstorm using Stille and Suzuki reactions for PET radiopharmaceuticals. Largely driven by mild conditions as opposed to the harsh conditions of conventional fluorine labeling, high radiochemical yields and fast kinetics, the metal-mediated cross-coupling reactions are being increasingly validated. The review presented by Doi (80) and Pretze et al. (82), cover the historical and development details for Stille, Suzuki coupling, Negishi coupling, and Sonogashira and Heck coupling. Among the reactions, Stille reaction has been widely applied in the synthesis of radiopharmaceuticals.

**Figure 7:**
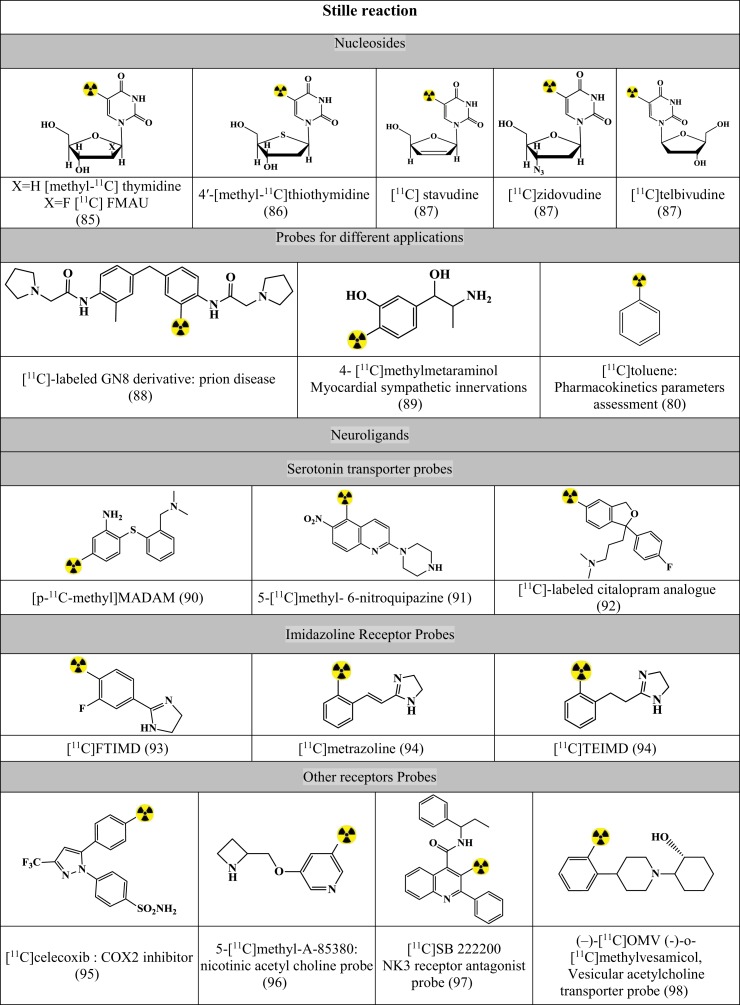

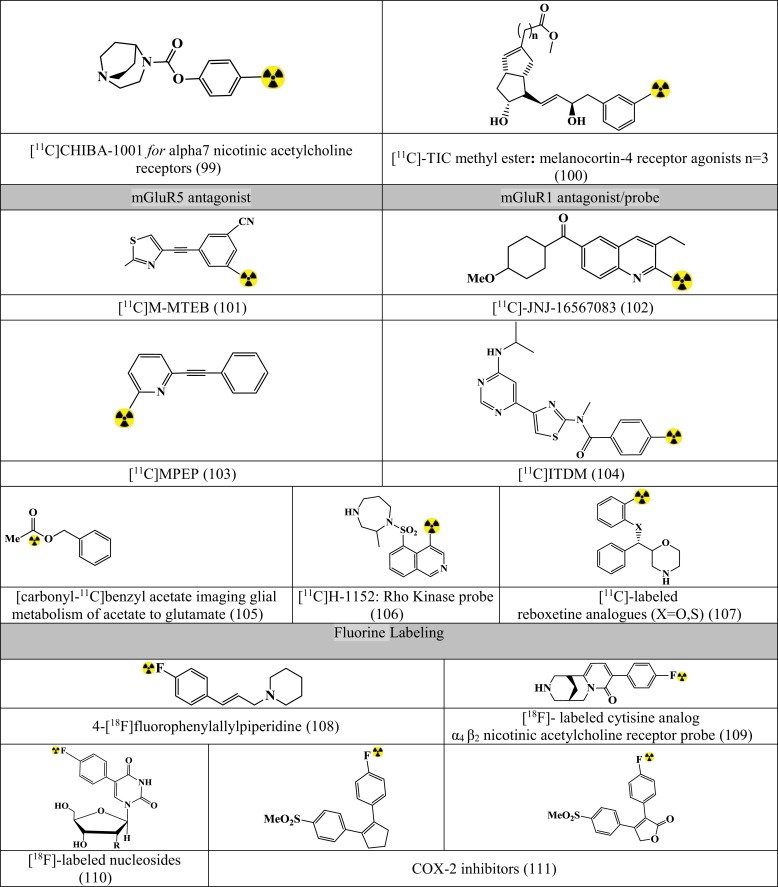
**Selected structures developed using Stille reaction**. Structures referenced in (80, 82, 85–111).

**Figure 8:**
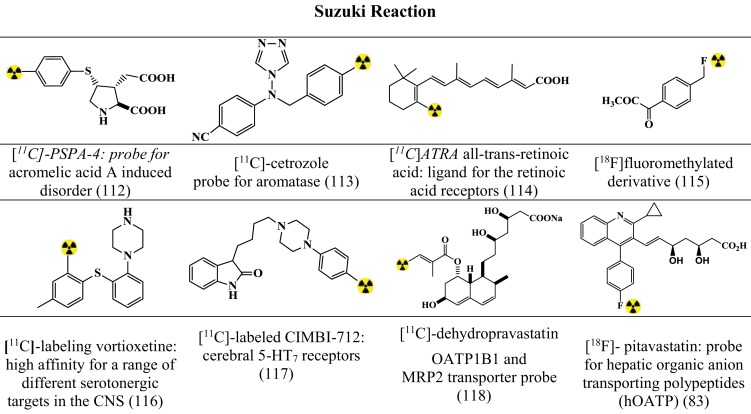
**Selected structures developed using Suzuki reactions**. Structures referenced in (80, 82, 83, 112–118).

**Figure 9:**
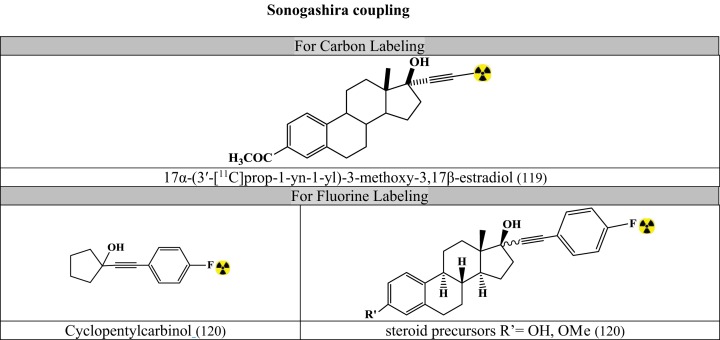
**Selected radiopharmaceuticals developed using Sonogashira coupling**. Structures referenced in (80, 82, 119–120).

Figures 7–9 summarizes the major contribution of the cross-coupling reactions in the development of radiopharmaceuticals.

#### Stille Reaction

Stille reaction involves coupling between an organotin compound with alkyl or aryl halogenide using Pd-catalyst and a phosphane-based coligand for the formation of both C–C bond and C–X bond (80, 82). The reaction has been tested with the following reaction conditions and validated for the synthesis of precursors as in Figure 7 (80, 82, 85–111).

Optimized conditions for catalyst and solvents include: (80, 82)

(Temperature and time dependent on reactants)

#### [^11^C]-Labeling

[^11^C]*-*labeled methyl iodide

- aromatictrimethylstannyl compounds in DMF or DMSO, Pd_2_(dba)_3_ with P(o-Tol)_3_ as coligand and CuCl/K_2_CO_3_ as additive in DMF
- aromatictrimethylstannyl compounds with Pd_2_(dba)_3_/P(o-Tol)_3_, DMF

carbonylative[^11^C]-CO coupling

- organic iodides with organostannanes in DMSO with an *e*xcess of P(o-Tol)_3_ relative to Pd-catalyst

[^11^C]-acetyl chloride

- organostannate with Pd_2_(dba)_3_ and coligand 2,8,9-trimethyl-2,5,8,9-tetraaza-1-phosphabicyclo[3.3.3]undecane hydrochloride in the ratio 1:0.5, respectively.

#### [^18^F]-Fluorination

1-[^18^F]fluoro-4-iodobenzene

- hexamethylphosphoramide is used as solvent and Pd(PPh_3_)_4_ as catalyst
- Pd(PPh_3_)_2_Cl_2_/CuI as catalyst in dioxane
- DMF/dioxane (1:1) or THF/dioxane (1:1) mixture using Pd_2_(dba)_3_/CuI/AsPh_3_
- Pd_2_(dba)_3_/P(o-Tol)_3_/CuI, DMF/toluene (1:1)

1-[^18^F]fluoro-4-bromobenzene:

- Pd_2_dba_3_/AsPh_3_ as mediator in a DMF:dioxane mixture (1:1)
- DMF/dioxane mixture and Pd(PPh_3_)_4_
- dioxane with PdCl(PPh_3_)_2_

Common conditions: DMF:dioxane mixture (1:1) as solvent and BnClPd(PPh_3_)_2_:CuI (ratio 1:1) as catalyst.

Advantages are (a) mild conditions, (b) wide tolerance of functional groups such as amino, hydroxyl, thiol, or carboxylate, and (c) stability of the organotin compounds.

Disadvantages include (a) metal linked toxicity and (b) kinetic and thermodynamic feasibility of the reaction.

Challenges are possible side reactions with different functional groups, difficult preparation and purification of stannyl compounds, reproducibility can be sensitive to the purification level of ^11^C-methyl iodide.

#### Suzuki Reactions

The Suzuki coupling is based on the conjugation of boron substrates (alkylborane/benzylborane/alkenylboranes) with alkyl halide leading to C–C bond formation or C–X bond formation (80, 82). General Optimized conditions (80, 82) for the synthesis of various precursors (Figure 8) include:

(Temperature and time dependent on reactants)

#### [^11^C]-Carbon Labeling

[^11^C]-methyl iodide

(i) aryl iodide or aryl boranes (reactant) with Pd(PPh_3_)_4_ as catalyst with THF as solvent under basic conditions.
(ii) aryl boranes (especially consisting acidic protons) in the presence of [Pd(dppf)Cl_2_] and K_3_PO_4_ in DMF under microwave heating
(iii) aryl boranes using Pd0-mediated conventional thermal heating method
(iv) pinacolphenylboronate/alkenylboranes/aryl boranewith Pd_2_(dba)_3_/P(o-tolyl)_3_/K_2_CO_3_ (1:4:4) in DMF or DMF/H_2_O (9:1)

[^11^C]-CO

(i) aryl iodides and phenylboronic acid (reactant) with Pd(PPh_3_)_2_Cl_2_(catalyst), K_2_CO_3_(base) and DMSO (solvent)
(ii) aryltriflate + alkyl boronic acid (reactant) with bases such as *tetra*-butylammonium fluoride or aryltriflate + aromatic boronic acid (reactant) with bases such as potassium *tert*-butoxide. Lithium bromide (promoter) may also be added.

#### Fluorine Labeling

[^18^F] fluoromethyl iodide ([^18^F]-FCH_2_I)

(i) pinacolphenylboronate with 1:3 ratio of Pd/P(o-tolyl)_3_

1-[^18^F]fluoro-4-iodobenzene

(i) organoboranes with Pd_2_(dba)_3_ as mediator, Cs_2_CO_3_ as base and acetonitrile as solvent.

Advantages are (a) borane derivatives that are less toxic than the stannous substrates, (b) organoborane has relatively high reactivity, especially in the presence of a base or a fluoride anion, (c) compatible with a wide variety of functionalities, and (d) water tolerant.

#### Sonogashira Coupling

Based on organocopper species that interacts with the Pd-catalyst in transmetalation step for conjugation of terminal alkynes with vinylic or aryl halides (Figure 9) (80, 82, 119–120).

Optimized conditions: (80, 82)

(Temperature and time dependent on reactants)

#### Carbon Labeling

[^11^C]-methyl iodide

- terminal alkyne with Pd_2_(dba)_3_, AsPh_3_ and tetra-*n*-butyl-ammonium fluoride in THF (for Sonogashira-like coupling)

#### Fluorine Labeling

- 4-[^18^F]fluoro-1-iodobenzene: THF as solvent and Et_3_N as base

#### Heck Reaction

**Figure 10:**
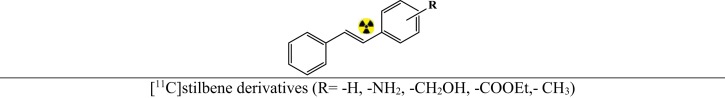
**Radiopharmaceutical developed using Heck reaction**. Structures referenced in (82, 121).

Based on palladium-catalyzed C–C bond formation between olefins and aryl/vinyl halides (Figure 10) (82).

#### Negishi Reaction and Misc Reactions

Negishi coupling can be a coupling of choice when other couplings fail (80, 82). It is based on organozincs as nucleophiles and is palladium-catalyzed reaction. Disadvantages include (a) incompatible with common functional groups, such as hydroxyl, sulfhydryl, aldehyde, and carboxylic acid, hence limited scope and (b) sensitive to environmental conditions.

#### Carbon Labeling: (Temperature and Time Dependent on Reactants)

Arylzinc iodide and 11C labeled methyl iodide with Pd(PPh_3_)Cl_2_ in dimethylacetamide at room temperature or elevated temperature.

**Figure 11:**
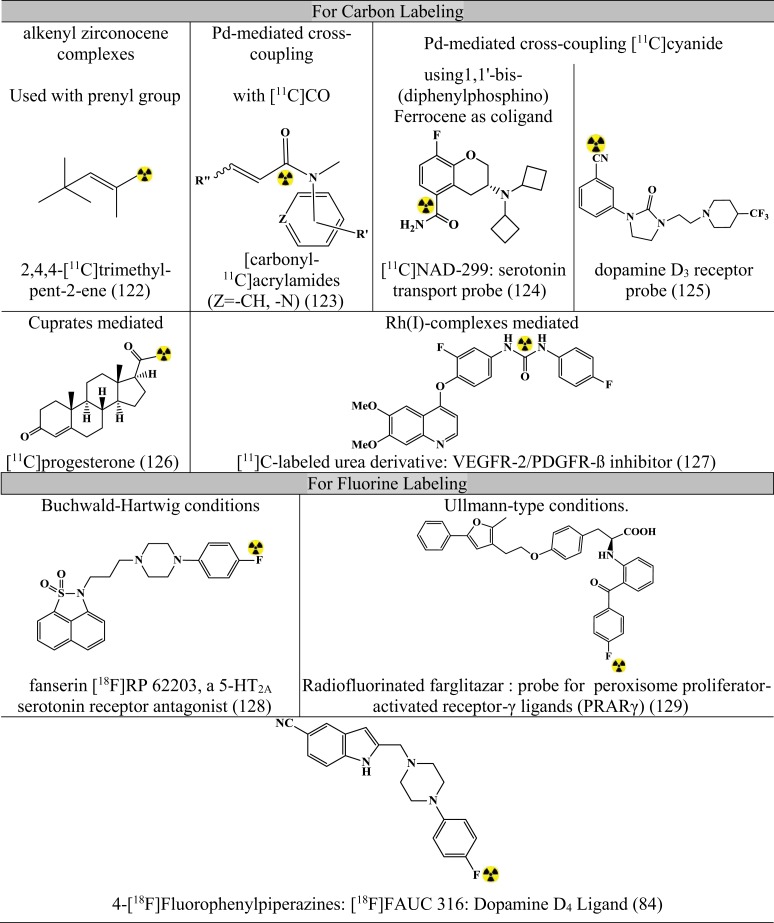
**Representative radiopharmaceuticals developed using Misc reactions**. Reference: (80, 82, 84, 122–129).

Apart from the above-mentioned cross-coupling reactions, there exist many miscellaneous reactions that can make an important contribution in near future. Figure 11 summarizes some contributions (80, 82, 84, 122–129).

##### Future Directions

The future directions for successfully utilizing the novel chemistries include (a) easy synthesis of precursors for example cyclooctynes and tetrazines (b) better purification techniques to remove metal linked toxic species, especially using cartridges or scavenger resins that allow faster and easy purification (c) standardization of novel approaches toward automated synthesis (d) regioisomer selectivity, and (e) studies to understand the effect of bulky precursors on pharmacokinetics and biological efficacy of radiopharmaceuticals.

## Conclusion

This review has summarized the applications and scope of the three approaches for the development of radiopharmaceuticals (a) bivalent ligand approach (BLA) for the novel design of the radiopharmaceuticals, (b) lipidization and surface modification, and (c) novel chemistries for radiolabeling. Despite the rise to prominence only 5–10 years ago all the above approaches have made a significant impact in radiolabeling and development of radiopharmaceuticals. The reactions have been tested with a wide variety of biomolecules-small molecules, steroids, nucleosides, glucose derivatives, peptides, and also with NPs. Selectivity, orthogonality, and fast kinetics are the key requirements for being a method of choice of novel chemistries.

## Author Contributions

All authors listed, have made substantial, direct and intellectual contribution to the work, and approved it for publication.

## Conflict of Interest Statement

The authors declare that the research was conducted in the absence of any commercial or financial relationships that could be construed as a potential conflict of interest.
